# Dopamine Signaling in reward-related behaviors

**DOI:** 10.3389/fncir.2013.00152

**Published:** 2013-10-11

**Authors:** Ja-Hyun Baik

**Affiliations:** Molecular Neurobiology Laboratory, Department of Life Sciences, Korea UniversitySeoul, South Korea

**Keywords:** dopamine, dopamine receptor, drug addiction, food reward, reward circuit

## Abstract

Dopamine (DA) regulates emotional and motivational behavior through the mesolimbic dopaminergic pathway. Changes in DA mesolimbic neurotransmission have been found to modify behavioral responses to various environmental stimuli associated with reward behaviors. Psychostimulants, drugs of abuse, and natural reward such as food can cause substantial synaptic modifications to the mesolimbic DA system. Recent studies using optogenetics and DREADDs, together with neuron-specific or circuit-specific genetic manipulations have improved our understanding of DA signaling in the reward circuit, and provided a means to identify the neural substrates of complex behaviors such as drug addiction and eating disorders. This review focuses on the role of the DA system in drug addiction and food motivation, with an overview of the role of D1 and D2 receptors in the control of reward-associated behaviors.

## INTRODUCTION

Dopamine (DA) is the predominant catecholamine neurotransmitter in the brain, and is synthesized by mesencephalic neurons in the substantia nigra (SN) and ventral tegmental area (VTA). DA neurons originate in these nuclei and project to the striatum, cortex, limbic system and hypothalamus. Through these pathways, DA affects many physiological functions, such as the control of coordinated movements and hormone secretion, as well as motivated and emotional behaviors (Hornykiewicz, 1966; Beaulieu and Gainetdinov, 2011; Tritsch and Sabatini, 2012).

Regulation of the DA system in reward-related behaviors has received a great deal of attention because of the serious consequences of dysfunction in this circuit, such as drug addiction and food reward linked obesity, which are both major public health issues. It is now well accepted that following repeated exposure to addictive substances, adaptive changes occur at the molecular and cellular level in the DA mesolimbic pathway, which is responsible for regulating motivational behavior and for the organization of emotional and contextual behaviors (Nestler and Carlezon, 2006; Steketee and Kalivas, 2011). These modifications to the mesolimbic pathway are thought to lead to drug dependence, which is a chronic, relapsing disorder in which compulsive drug-seeking and drug-taking behaviors persist despite serious negative consequences (Thomas et al., 2008). Recent findings suggest that glutamatergic and GABAergic synaptic networks in the limbic system are also affected by drugs of abuse, and that this can alter the behavioral effects of addictive drugs (Schmidt and Pierce, 2010; Lüscher and Malenka, 2011). Considerable evidence now suggests that substantial synaptic modifications of the mesolimbic DA system are associated with not only the rewarding effects of psychostimulants and other drugs of abuse, but also with the rewarding effects of natural reward, such as food; however, the mechanism by which drugs of abuse induce the modify synaptic strength in this circuit remains elusive. In fact, DA reward signaling seems extremely complex, and is also implicated in learning and conditioning processes, as evidenced by studies revealing a DAergic response coding a prediction error in behavioral learning, for example (Wise, 2004; Schultz, 2007, 2012), thus suggesting a need for a fine dissection at a circuit level to properly understand these motivated reward-related behaviors. Recent studies using optogenetics and neuron-specific or circuit-specific genetic manipulations are now allowing a better understanding of DA signaling in the reward circuit.

In this review, I will provide a short summary of DA signaling in reward-related behaviors, with an overview of recent studies on cocaine-addiction behaviors as well as some on food reward in the context of the role of D1 and D2 receptors in regulating these behaviors.

## DOPAMINE RECEPTORS

Dopamine interacts with membrane receptors belonging to the family of seven transmembrane domain G-protein coupled receptors, with activation leading to the formation of second messengers, and the activation or repression of specific signaling pathways. To date, five different subtypes of DA receptors have been cloned from different species. Based on their structural and pharmacological properties, a general subdivision into two groups has been made: the D1-like receptors, which stimulate intracellular cAMP levels, comprising D1 (Dearry et al., 1990; Zhou et al., 1990) and D5 (Grandy et al., 1991; Sunahara et al., 1991), and the D2-like receptors, which inhibit intracellular cAMP levels, comprising D2 (Bunzow et al., 1988; Dal Toso et al., 1989), D3 (Sokoloff et al., 1990), and D4 (Van Tol et al., 1991) receptors.

D1 and D2 receptors are the most abundantly expressed DA receptors in the brain. The D2 receptor has two isoforms generated by alternative splicing of the same gene (Dal Toso et al., 1989; Montmayeur et al., 1991). These isoforms, named D2L and D2S, are identical except for an insert of 29 amino acids present in the putative third intracellular loop of D2L, an intracellular domain thought to play a role in coupling this class of receptor to specific second messengers.

D2 receptors are localized presynaptically, revealed by D2 receptor immunoreactivity, mRNA, and binding sites present in DA neurons throughout the midbrain (Sesack et al., 1994), with lower level of D2 receptor expression in theVTA than in the SN (Haber et al., 1995). These D2-type autoreceptors represent either somatodendritic autoreceptors, known to dampen neuronal excitability (Lacey et al., 1987, 1988; Chiodo and Kapatos, 1992), or terminal autoreceptors, which mostly decrease DA synthesis and packaging (Onali et al., 1988; Pothos et al., 1998), but also inhibit impulse-dependent DA release (Cass and Zahniser, 1991; Kennedy et al., 1992; Congar et al., 2002). Therefore, the principal role of these autoreceptors is the inhibition and modulation of overall DA neurotransmission; however, it has been suggested that in the embryonic stage, the D2-type autoreceptor could have a different function in DA neuronal development (Kim et al., 2006, 2008; Yoon et al., 2011; Yoon and Baik, 2013). Thus, the cellular and molecular role of these presynaptic D2 receptors needs to be explored further. The expression of D3, D4, and D5 receptors in the brain is considerably more restricted and weaker than that of either D1 or D2 receptors.

There is some difference in the affinity of DA for D1-like receptors and D2-like receptors, mostly reported on the basis of receptor-ligand binding assay studies using heterologously expressed DA receptors in cell lines. For example, D2-like receptors seem to have a 10- to 100-fold greater affinity for DA than the D1-like family, with the D1 receptor reported to have the lowest affinity for DA (Beaulieu and Gainetdinov, 2011; Tritsch and Sabatini, 2012). These differences suggest a differential role for the two receptors given that DA neurons can have two different patterns of DA release, “tonic” or “phasic” based on their firing properties (Grace et al., 2007). It has been suggested that low-frequency, irregular firing of DA neurons tonically generates a low basal level of extracellular DA (Grace et al., 2007), while burst firing, or “phasic” activity is crucially dependent on afferent input, and is believed to be the functionally relevant signal sent to postsynaptic sites to indicate reward and modulate goal-directed behavior (Berridge and Robinson, 1998; Schultz, 2007; Grace et al., 2007). Therefore, bursting activity of DA neurons, leading to a transient increase in the DA level, is thought to be a key component of the reward circuitry (Overton and Clark, 1997; Schultz, 2007). Consequently, the D1 receptor, which is known as the low-affinity DA receptor, is thought to be preferentially activated by the transient, high concentrations of DA mediated by phasic bursts of DA neurons (Goto and Grace, 2005; Grace et al., 2007). In contrast, it is hypothesized that D2-like receptors, which are known to have a high affinity for DA, can detect the lower levels of tonic DA release (Goto et al., 2007). However, given that measurements of receptor affinity rely on ligand binding assays from heterologously expressed DA receptors, and do not reflect the receptor’s coupling capacity to downstream signaling cascades, it is difficult to infer whether D2-like receptors are preferentially activated by basal extracellular levels of DA *in vivo*. Thus, it remains to be elucidated how these two different receptors participate in different pattern of DA neuronal activity *in vivo*.

## SIGNALING PATHWAYS MEDIATED BY D1 AND D2 RECEPTORS

The D1- and D2-like receptor classes differ functionally in the intracellular signaling pathways they modulate. The D1-like receptors, including D1 and D5, are coupled to heterotrimeric G-proteins that include the G proteins Gα_s_ and Gα_olf_, with activation leading to increased adenylyl cyclase (AC) activity, and increased cyclic adenosine monophosphate (cAMP) production. This pathway induces the activation of protein kinase A (PKA), resulting in the phosphorylation of variable substrates and the induction of immediate early gene expression, as well as the modulation of numerous ion channels. In contrast, D2-class DA receptors (D2, D3, and D4) are coupled to Gα_i_ and Gα_o_ proteins, and negatively regulate the production of cAMP, resulting in decreased PKA activity, activation of K^+^ channels, and the modulation of numerous other ion channels (Kebabian and Greengard, 1971; Kebabian and Calne, 1979; Missale et al., 1998; Beaulieu and Gainetdinov, 2011).

One of best-studied substrates of PKA is the DA- and cAMP-regulated phosphoprotein, Mr ~32,000 (DARPP-32), which is an inhibitor of protein phosphatase, and is predominantly expressed in medium spiny neurons (MSNs) of the striatum (Hemmings et al., 1984a). It appears that DARPP-32 acts as an integrator involved in the modulation of cell signaling in response to DA in striatal neurons. It has been demonstrated that phosphorylation of DARPP-32 at threonine 34 by PKA activates inhibitory function of DARPP-32 over the protein phosphatase (PP1; Hemmings et al., 1984a,b). In D1 receptor expressing striatal neurons, D1 receptor stimulation results in an increased phosphorylation of DARPP-32 in response to PKA activation, while stimulation of D2 receptors in D2 receptor-expressing neurons reduces the phosphorylation of DARPP-32 at threonine 34, presumably as a consequence of reduced PKA activation (Bateup et al., 2008). However, it appears that a cAMP-independent pathway also participates in the D2-receptor-mediated regulation of DARPP-32, given that dephosphorylation of threonine 34 by the calmodulin-dependent protein phosphatase 2B (PP2B; also known as calcineurin), which is activated by increased intracellular Ca^2^^+^following D2 receptor activation (Nishi et al., 1997). These findings suggest that DA exerts a bidirectional control on the state of phosphorylation of DARPP-32, a DA-centered signaling molecule. Therefore, one can imagine that overall, under DA tone, these signaling pathways mediated by the two classes of receptors can influence neuronal excitability, and consequently synaptic plasticity, in terms of their synaptic networks in the brain, given that their precise signaling varies depending on the cell type and brain region in which they are expressed (Beaulieu and Gainetdinov, 2011; Girault, 2012).

In the case of D2 receptors, the situation is further complicated, as D2 receptors are alternatively spliced, giving rise to isoforms with distinct physiological properties and subcellular localizations. The large isoform appears to be expressed dominantly in all brain regions, although the exact ratio of the two isoforms can vary (Montmayeur et al., 1991). In fact, the phenotype of D2 receptor total knockout (KO) mice was found to be quite different from that of D2L KO mice (Baik et al., 1995; Usiello et al., 2000), indicating that the two isoformsmight have different functions *in vivo*. Recent results from Moyer et al. (2011) support a differential *in vivo* function of the D2 isoforms in human brain, showing a role of two variants of D2 receptor gene with intronic single-nucleotide polymorphisms (SNPs) in D2 receptor alternative splicing, and a genetic association between these SNPs and cocaine abuse in Caucasians (Moyer et al., 2011; Gorwood et al., 2012).

## DA-MEDIATED SIGNALING IN ACTIVATION OF MITOGEN-ACTIVATED PROTEIN KINASES

One signaling pathway of particular interest in neurons is the mitogen-activated protein kinases, extracellular-signal regulated kinases (ERK), which are activated by D1 and D2 receptors. It is now widely accepted that ERK activation contributes to different physiological responses in neurons, such as cell death and development, as well as synaptic plasticity, and that modulating ERK activity in the CNS can result in different neurophysiological responses (Chang and Karin, 2001; Sweatt, 2004; Thomas and Huganir, 2004). Additionally, ERK activation can be regulated by various neurotransmitter systems, a process that can be complex but is finely tuned depending on the differential regulation of the signaling pathways mediated by the various neurotransmitters. Therefore, it is interesting to see what the physiological output of ERK signaling upon DA stimulation through these receptors would be.

**FIGURE 1:**
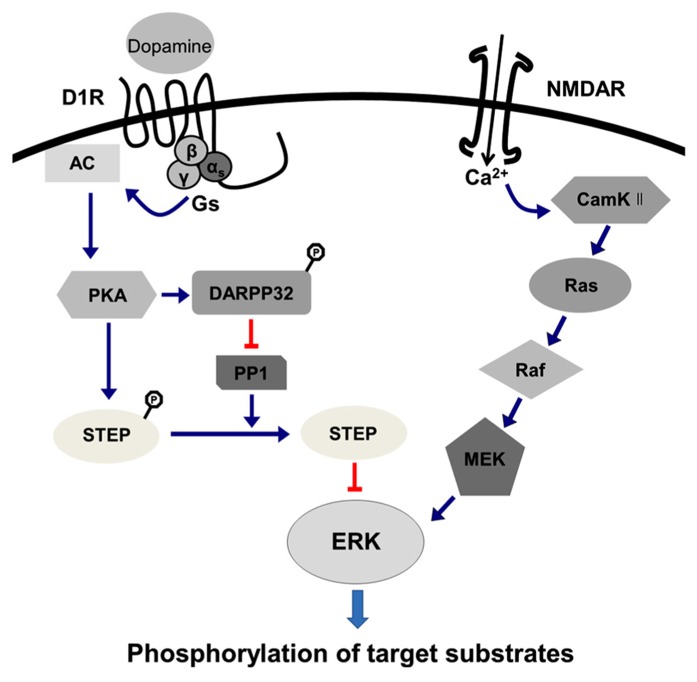
**D1 receptor-mediated ERK activation signaling pathway.** D1 receptor-mediated ERK singling involves interaction with the NMDA glutamtate receptor (see text), which is expressed predominantly in the striatum. The stimulation of D1 receptors is not able to mediate ERK phosphorylation per se, but rather requires endogenous glutamate (Pascoli et al., 2011). Stimulation of D1 receptors increases calcium influx through NMDA receptors, which involves phosphorylation of the NMDA receptor NR2B subunit by a Src-family tyrosine kinase (Pascoli et al., 2011). This increased calcium influx activates a number of signaling pathways, including calcium and calmodulin-dependent kinase II (CamKII), which can activate ERK via the Ras-Raf-MEK cascade (Fasano et al., 2009; Shiflett and Balleine, 2011; Girault, 2012). Upon D1 receptor activation, activated PKA can mediate phosphorylation of DARPP-32 and phosphorylated DARPP-32 can act as potent inhibitor of the protein phosphatase (PP-1), which dephosphorylates another phosphatase, the striatal-enriched tyrosine phosphatase (STEP). Dephosphorylation of STEP activates its phosphatase activity, thus allowing STEP to dephosphorylate ERK. DARPP-32 also acts upstream of ERK, possibly by inhibiting PP-1, which prevents PP-1 from dephosphorylating MEK, the upstream kinase of ERK. Thus, D1 receptor activation increases ERK phosphorylation by preventing its dephosphorylation by STEP, but also by preventing the dephosphorylation of the upstream kinase of ERK, indicating that D1 receptor-mediated ERK activation involved a complex regulation by phosphatases and kinases in addition to the cross talk with glutamate receptor signaling. Phosphorylation status is only notified for DARPP32 and STEP in this figure.

Results obtained from heterologous cell culture systems suggest that both D1- and D2-class DA receptors can regulate ERK1 and 2 (Choi et al., 1999; Beom et al., 2004; Chen et al., 2004; Kim et al., 2004; Wang et al., 2005). D1 receptor-mediated ERK singling involves an interaction with the NMDA glutamtate receptor (Valjent et al., 2000, 2005), which has been mostly described in the striatum. D1 receptor stimulation is not able to mediate ERK phosphorylation in itself, but rather requires endogenous glutamate (Pascoli et al., 2011). With D1 receptor activation, activated PKA can mediate the phosphorylation of DARPP-32 at its Thr-34, as mentioned above. Phosphorylated DARPP-32 can act as potent inhibitor of the protein phosphatase PP-1, which dephosphorylates another phosphatase, the striatal-enriched tyrosine phosphatase (STEP). Dephosphorylation of STEP activates its phosphatase activity, thus allowing STEP to dephosphorylate ERK (Paul et al., 2003). DARPP-32 also acts upstream of ERK, possibly by inhibiting PP-1, preventing PP-1 from dephosphorylating MEK, the upstream kinase of ERK (Valjent et al., 2005). Thus, D1 receptor activation acts to increase ERK phosphorylation by preventing its dephosphorylation by STEP, but also by preventing the dephosphorylation of the upstream kinase of ERK. In addition, the cross talk between D1 and NMDA receptors contributes to the ERK activation. For example, a recent study showed that stimulation of D1 receptors increases calcium influx through NMDA receptors, a process that involves phosphorylation of the NMDA receptor NR2B subunit by a Src-family tyrosine kinase (Pascoli et al., 2011). This increased calcium influx activates a number of signaling pathways, including calcium and calmodulin-dependent kinase II, which can activate ERK via the Ras-Raf-MEK cascade (Fasano et al., 2009; Shiflett and Balleine, 2011; Girault, 2012). Consequently, D1 receptor-mediated ERK activation employs a complex regulation by phosphatases and kinases in addition to the cross talk with glutamate receptor signaling (**Figure 1**).

**FIGURE 2:**
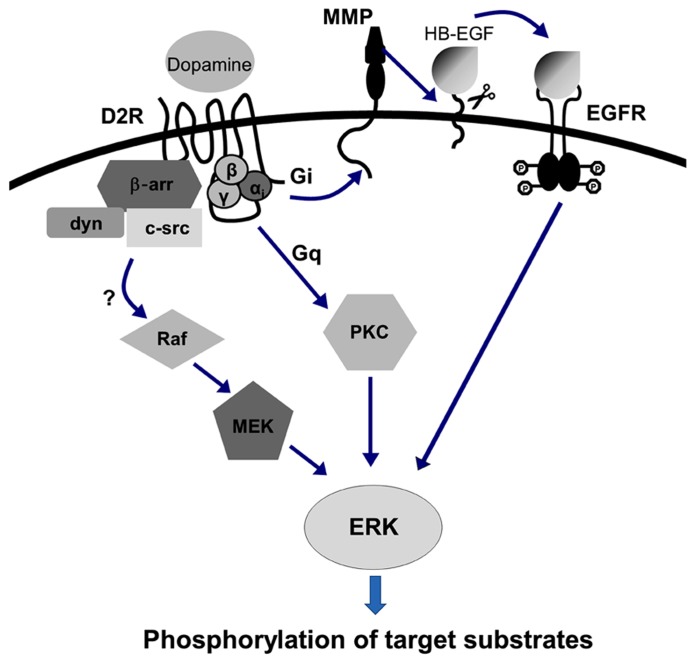
**D2 receptor-mediated ERK activation signaling pathway.** D2 receptor-mediated ERK activation is dependent on Gα_i_ protein coupling. It also appears that D2 receptor-mediated ERK activation requires the transactivation of receptor tyrosine kinase, which consequently activates downstream signaling involving matrix metalloproteinases (MMPs) with ectodomain shedding of EGFR ligand, for example, to finally activate ERK (Choi et al., 1999; Kim et al., 2004; Wang et al., 2005; ). The involvement of arrestin has also been suggested to contribute to D2 receptor-mediated ERK activation (Beom et al., 2004; Kim et al., 2004), which can activate MAPK signaling by mobilizing clathrin-mediated endocytosis in a β-arrestin/dynamin-dependent manner (Kim et al., 2004).

D2 receptor-mediated ERK activation has been reported in heterologous cell culture systems (Luo et al., 1998; Welsh et al., 1998; Choi et al., 1999). D2 receptor-mediated ERK activation was found to be dependent on Gα_i_ protein coupling, and it appears thatit requires the transactivation of receptor tyrosine kinase, which activates downstream signaling to finally activate ERK (Choi et al., 1999; Kim et al., 2004; Wang et al., 2005; Yoon et al., 2011; Yoon and Baik, 2013). Arrestin has been also suggested to contribute to D2 receptor-mediated ERK activation (Beom et al., 2004; Kim et al., 2004), which can activate MAPK signaling by mobilizing clathrin-mediated endocytosis in a β-arrestin/dynamin-dependent manner (Kim et al., 2004). A further possibility of D2 receptorscoupling to Gq proteins cannot be ruled out; in this case, Gq protein-mediated PKC activation could also induce ERK activation (Choi et al., 1999; **Figure 2**).

In view of the physiological role of this DA receptor-mediated ERK signaling, it has been shown that in mesencephalic neurons, DA activates ERK signaling via mesencephalic D2 receptors, which in turn activates the transcription factors such as Nurr1, a transcription factor critical for the development of DA neurons (Kim et al., 2006). Furthermore, our recent work demonstrated that STEP or Wnt5a can be involved in this regulation, by interacting with D2 receptors (Kim et al., 2008; Yoon et al., 2011). In light of these findings, it is intriguing whether this signaling can play a role in DA neurotransmission in the adult brain.

However, in the dorsal striatum, administration of the typical anti-psychotic D2-class receptor antagonist haloperidol stimulated the phosphorylation of ERK1/2, while the atypical anti-psychotic clozapine, which is also a D2-class antagonist, reduced ERK1/2 phosphorylation, showing that haloperidol and clozapine induce distinct patterns of phosphorylation in the dorsal striatum (Pozzi et al., 2003). Thus, the physiological relevance of this D2 receptor-mediated ERK signaling remains as an open issue.

Taken together, it is evident that D1and D2 receptors induce ERK activation via distinct mechanisms, and one can imagine that activation of these receptors can have different consequences, depending on the location and physiological status of the neurons expressing them.

## ROLE OF D1 AND D2 RECEPTORS IN DRUG INDUCED BEHAVIORS

The role of D1 and D2 receptors in reward-related behaviors has been investigated pharmacologically using subtype specific agonists and antagonists, as well as by the analysis of receptor gene KO mice. Recent progress in optogenetics and the use of viral vectors with different genetic manipulations now allow a refined examination of the functional importance of these receptors *in vivo* (**Table 1**).

**Table 1:** Role of dopamine D1 and D2 receptors in cocaine-induced behaviors.

Cocaine-induced behaviors	Receptor-type	Animal models	Effects mediated by cocaine	Reference
Cocaine-induced locomotor activity	D1	D1 antagonist SCH23390	Diminished locomotor response to cocaine	Cabib et al. (1991), Ushijima et al. (1995), Hummel and Unterwald (2002)
	D1	D1 KO mice	Diminished locomotor response	Xu et al. (1994)
	D1	Loss of DARPP 32 in D1 cell, using DARPP 32 flox mice x D1-Cre mice	Diminished acute locomotor response to cocaine	Bateup (2010)
	D2	D2 antagonist Haloperidol, raclopride	Unaffected	Cabib et al. (1991), Ushijima et al. (1995)
	D2	D2 KO mice	Increased but low level	Chausmer et al. (2002), Welter et al. (2007), Sim et al. (2013)
	D2	Loss of DARPP 32 in D2 cell, using DARPP 32 flox mice x D2-Cre mice	Increased acute locomotor response to cocaine	Bateup (2010)
Cocaine-induced locomotor sensitization	D1	D1 antagonist SCH23390 (i.p or VTA)	Unaffected	Kuribara and Uchihashi (1993), Mattingly et al. (1994), Steketee (1998), White et al. (1998), Vanderschuren and Kalivas (2000)
	D1	D1 KO mice	Not fully prevent cocaine sensitization at all doses	Karlsson et al. (2008)
	D1	Optogenetically activated with Conditional ChR2 viruses injected in NAc of D1-Cre mice	Enhanced cocaine sensitization	Lobo (2010)
	D1	Inhibition of D1 cell with Tetanus toxin light chain expression in D1-MSNs	Diminished cocaine sensitization	Hikida et al. (2010)
	D1	Optogenetic inactivation of D1-MSN cells with halorhodopsin	Diminished cocaine sensitization	Chandra et al. (2013)
	D1	Reconstruction of D1 expression in NAc of D1 KO mice	Mediate D1-dependent cocaine sensitization	Gore and Zweifel (2013)
	D2	D2 antagonist sulpride, YM-09151-2, eticlopride,	Unaffected	Kuribara and Uchihashi (1993), Mattingly et al. (1994), Vanderschuren and Kalivas (2000)
	D2	D2 agonist quinpirole in intra-medial prefrontal cortex	Blunted cocaine sensitization	Beyer and Steketee (2002)
	D2	D2 KO mice	Unaffected	Sim et al. (2013)
	D2	Depletion of D2 receptors in the NAc by shRNA delivery	Unaffected	Sim et al. (2013)
	D2	Optogenetically activated with Conditional ChR2 viruses injected in NAc of D2-Cre mice	Unaffected	Lobo (2010)
	D2	Inhibition of D2 cell with Tetanus toxin light chain expression in D2-MSNs	Slightly decreased cocaine sensitization	Hikida et al. (2010)
CPP	D1	Systemic and intra-accumbens administration of the D1 receptor antagonist SCH23390	Diminished cocaine CPP	Cervo and Samanin (1995), Baker et al. (1998)
	D1	D1 KO mice	Normal CPP	Miner et al. (1995), Karasinska et al. (2005)
	D1	Optogenetically activated with Conditional ChR2 viruses injected in NAc of D1-Cre mice	Enhanced cocaine CPP	Lobo (2010)
	D1	Inhibition of D1 cell with Tetanus toxin light chain expression in D1-MSNs	Diminished cocaine CPP	Hikida et al. (2010)
	D2	Systemic administration of the D2 receptor antagonist	Normal CPP	Spyraki et al. (1982), Cervo and Samanin (1995), Shippenberg and Heidbreder (1995), Nazarian et al. (2004)
	D2	D2 KO mice	Normal CPP	Welter et al. (2007), Sim et al. (2013)
	D2	Depletion of D2 receptors in the NAc by shRNA delivery	Normal CPP	Sim et al. (2013)
	D2	Optogenetically activated with Conditional ChR2 viruses injected in NAc of D2-Cre mice	Diminished cocaine CPP	Lobo (2010)
	D2	Inhibition of D2 cell with Tetanus toxin light chain expression in D2-MSNs	No change in CPP	Hikida et al. (2010)
	D2	Conditional KO of D2 autoreceptors	Enhanced cocaine CPP	Bello et al. (2011)
Cocaine self- administration and cocaine-seeking behaviors	D1	D1 antagonist	Dose-dependent bipasic effect in self-administration	Woolverton (1986), Britton et al. (1991), Hubner and Moreton (1991), Vanover et al. (1991), Caine and Koob (1994)
	D1	D1 agonist	No effect in reinstatement of cocaine-seeking behavior	Self et al. (1996), De Vries et al. (1999), Spealman et al. (1999), Khroyan et al. (2000)
	D1	D1 KO mice	Elimiated cocaine self-administration	Caine et al. (2007)
	D2	D2 antagonist	Dose-dependent biphasic effect in self-administration	Woolverton (1986), Britton et al. (1991), Hubner and Moreton (1991), Caine and Koob (1994)
	D2	D2 agonist	Induce reinstatement of cocaine-seeking behavior	Self et al. (1996), De Vries et al. (1999),^2002^, Spealman et al. (1999), Khroyan et al. (2000), Fuchs et al. (2002)
	D2	D2 KO mice	Increased cocaine self-administration	Caine et al. (2002)
	D2	D2 KO mice	With stress, reinstatement of cocaine-seeking behavior is attenuated	Sim et al. (2013)
	D2	Optogenetic activation of D2-MSNs in NAc	Suppress cocaine self-administration	Bock et al. (2013)

### COCAINE-INDUCED BEHAVIORAL SENSITIZATION

Exposure to a psychostimulant such as cocaine induces a progressive and enduring enhancement in the locomotor stimulant effect of subsequent administration, a phenomenon known as sensitization (Robinson and Berridge, 1993; Vanderschuren and Kalivas, 2000; Kalivas and Volkow, 2005; Steketee and Kalivas, 2011). The process of behavioral sensitization includes two distinct phases; initiation and expression. The initiation phase refers to the period during which the increased behavioral response following daily cocaine administration is associated with an increase in extracellular DA concentration. Behavioral sensitization continues to increase after the cessation of cocaine administration, and this procedure produces long-lasting sensitization, known as the expression of sensitization (Vanderschuren and Kalivas, 2000; Thomas et al., 2001; Steketee and Kalivas, 2011). The expression phase is characterized by a persistent drug hyper-responsiveness after cessation of the drug, which is associated with a cascade of neuroadaptation (Kalivas and Duffy, 1990; Robinson and Berridge, 1993). While this phenomenon has been studied mostly in experimental animals, the neuronal plasticity underlying behavioral sensitization is believed to reflect the neuroadaptations that contribute to compulsive drug cravings in humans (Robinson and Berridge, 1993; Kalivas et al., 1998). It has been suggested that the mesolimbic DA system from the VTA to the nucleus accumbens (NAc) and prefrontal cortex is an important mediator of these plastic changes, in association with the glutamatergic circuitry (Robinson and Berridge, 1993; Kalivas et al., 1998; Vanderschuren and Kalivas, 2000).

Animals behaviorally sensitized to cocaine, amphetamine, nicotine, or morphine (Kalivas and Duffy, 1990; Parsons and Justice, 1993) show enhanced DA release in the NAc in response to drug exposure. In addition to changes in neurotransmitter release, DA binding to its receptors plays a key role in behavioral sensitization (Steketee and Kalivas, 2011). For example, the enhanced excitability of VTA DA neurons that occurs with repeated cocaine exposure is associated with decreased D2 autoreceptor sensitivity (White and Wang, 1984; Henry et al., 1989). In addition, repeated intra-VTA injections of low doses of the D2 antagonist eticlopride, which is presumably autoreceptor-selective, enhanced subsequent responses to amphetamine (Tanabe et al., 2004).

A number of studies have shown that D1 and D2 DA receptors are differentially involved in cocaine-induced changes in locomotor activity. For example, initial studies employing pharmacological approaches have shown that mice or rats pre-treated with the D1 receptor antagonist SCH 23390 showed an attenuated locomotor response to acute cocaine challenge, while the D2 receptor antagonists haloperidol, and raclopride had no such effect (Cabib et al., 1991; Ushijima et al., 1995; Hummel and Unterwald, 2002). These results suggest different roles of DA receptor subtypes in the modulation of the stimulant effects of cocaine on locomotion. However, with regards to the behavioral sensitization induced by repetitive injections of cocaine, it has been reported that systemic administration of the D1 receptor antagonist SCH23390, or of the D2 receptor antagonists sulpiride, YM-09151-2 or eticlopride, does not affect the induction of cocaine sensitization (Kuribara and Uchihashi, 1993; Mattingly et al., 1994; Steketee, 1998; White et al., 1998; Vanderschuren and Kalivas, 2000).

The effects of direct intra-accumbens administration of SCH23390 on cocaine-induced locomotion, sniffing, and conditioned place preference (CPP) were investigated in rats, and these studies showed that the stimulation of D1-like receptors in the NAc is necessary for cocaine-CPP, but not for cocaine-induced locomotion (Baker et al., 1998; Neisewander et al., 1998). The direct intra-accumbens infusion of the D2/D3 receptor antagonist sulpiride in rats demonstrated that blockade of D2 receptors reverses the acute cocaine-induced locomotion (Neisewander et al., 1995; Baker et al., 1996), but these studies did not examine the effect on cocaine-induced behavioral sensitization. Interestingly, it has been reported that injection of the D2 receptor agonist quinpirole into the intra-medial prefrontal cortex blocked the initiation and attenuated the expression of cocaine-induced behavioral sensitization (Beyer and Steketee, 2002).

D1 receptor null mice have been examined in the context of addictive behaviors, and initial studies revealed that D1 receptor mutant mice failed to exhibit the psychomotor stimulant effect of cocaine on motor and stereotyped behaviors compared to their wild-type littermates (Xu et al., 1994; Drago et al., 1996). However, it appears that D1 receptor KO abolishes the acute locomotor response to cocaine, but does not fully prevent locomotor sensitization to cocaine at all doses (Karlsson et al., 2008), demonstrating that genetic KO of D1 receptors is not sufficient to fully block cocaine sensitization under all conditions.

In D2 receptor KO mice, with reduced general locomotor activity, the cocaine-induced motor activity level is low compared to WT mice, but these animals were similar in terms of the ability to induce cocaine-mediated behavioral sensitization, or cocaine-seeking behaviors with a slight decrease in sensitivity (Chausmer et al., 2002; Welter et al., 2007; Sim et al., 2013). Depletion of D2 receptors in the NAc by infusion of a lentiviral vector with a shRNA against the D2 receptor did not affect basal locomotor activity, nor cocaine-induced behavioral sensitization, but conferred stress-induced inhibition of the expression of cocaine-induced behavioral sensitization (Sim et al., 2013). These findings, together with previous reports, strongly suggest that blockade of D2 receptors in the NAc does not prevent cocaine-mediated behavioral sensitization, and that D2 receptor in the NAc play a distinct role in the regulation of synaptic modification triggered by stress and drug addiction.

Recent studies using genetically engineered mice that express Cre recombinase in cell-type specific manner, revealed some role of D1 or D2 receptor-expressing MSNs in cocaine-addictive behaviors. For example, loss of DARPP-32 in D2 receptor-expressing cells resulted in an enhanced acute locomotor response to cocaine (Bateup, 2010). Hikida and co-workers used AAV vectors to express tetracycline-repressive transcription factor (tTa) using substance P (for D1-expressing MSNs) or enkephalin (for D2-expressing MSNs) promoters (Hikida et al., 2010). These vectors were injected into the NAc of mice, in which tetanus toxin light chain (TN) was controlled by the tetracycline-responsive element, to selectively abolish synaptic transmission in each MSN subtype. Reversible inactivation of D1/D2 receptor-expressing MSNs with the tetanus toxin (Hikida et al., 2010) revealed the predominant roles of the D1 receptor-expressing cells in reward learning and cocaine sensitization, but there was no change in sensitization caused by the inactivation of D2 receptor-expressing cells. Using DREADD (designer receptors exclusively activated by a designer drugs) strategies, with viral-mediated expression of an engineered GPCR (G_i/o_-coupled human muscarinic M_4_DREADD receptor, hM_4_D) that is activated by an otherwise pharmacologically inert ligand, Ferguson et al. (2011) showed that the activation of striatal D2 receptor-expressing neurons facilitated the development of amphetamine-induced sensitization. However, the optogenetic activation of D2 receptor-expressing cells in the NAc induced no change in cocaine-induced behavioral sensitization (Lobo, 2010).

Optogenetic inactivation of D1 receptor-expressing MSNs using the light activated chloride pump, halorhodopsin eNpHR3.0 (enhanced *Natronomonas pharaonis* halorhodopsin 3.0), during cocaine exposure resulted in an attenuation of cocaine-induced locomotor sensitization (Chandra et al., 2013). Furthermore, the conditional reconstruction of functional D1 receptor signaling in subregions of the NAc in D1 receptor KO mice resulted in D1 receptor expression in the core region of the NAc, but not the shell, mediated D1 receptor-dependent cocaine sensitization (Gore and Zweifel, 2013). These findings suggest that DA mechanisms critically mediate cocaine-induced behavioral sensitization, with distinct roles for D1 and D2 receptors, although the precise contribution of D1 and D2 receptors and their downstream signaling pathways remains to be determined.

### CONDITIONED PLACE PREFERENCE

The CPP paradigm is a commonly used preclinical behavioral test with a classical (Pavlovian) conditioning model. During the training phase of CPP, one distinct context is paired with drug injections, while another context is paired with vehicle injections (Thomas et al., 2008). During a subsequent drug-free CPP test, the animal chooses between the drug- and the vehicle-paired contexts. An increased preference for the drug context serves as a measure of the drug’s Pavlovian reinforcing effects (Thomas et al., 2008).

Although it has been previously reported that both systemic and intra-accumbens administration of the D1 receptor antagonist SCH23390 prevented cocaine CPP (Cervo and Samanin, 1995; Baker et al., 1998), D1 receptor mutant mice have been reported to demonstrate normal responses to the rewarding effects of cocaine in the CPP paradigm (Miner et al., 1995; Karasinska et al., 2005). Regarding the role of D2 receptors in CPP, there is considerable consensus in the literature that D2-like antagonists fail to influence place preference induced by cocaine (Spyraki et al., 1982; Shippenberg and Heidbreder, 1995; Cervo and Samanin, 1995; Nazarian et al., 2004). Consistent with these pharmacological studies, D2 receptor KO mice displayed a comparable CPP score to WT mice (Welter et al., 2007; Sim et al., 2013). Furthermore, D2L-/- mice developed a CPP to cocaine as did WT mice (Smith et al., 2002).

Recently, the effect of a conditional presynaptic KO of D2 receptors on addictive behaviors has been reported, and this study demonstrated that mice lacking D2 autoreceptors displayed cocaine supersensitivity, exhibited increased place preference for cocaine, as well as enhanced motivation for food reward, perhaps owing to the absence of presynaptic inhibition by autoreceptors that further elevates extracellular DA and maximizes the stimulation of postsynaptic DA receptors (Bello et al., 2011).

Results obtained from a different line of investigation showed that when D1-expressing MSNs are selectively activated by optogenetics, D1-Cre mice expressing DIO-AAV-ChR2-EYFP in the NAc displayed a significant increase in cocaine/blue-light preference compared to the control group (Lobo, 2010). In contrast, D2-Cre mice expressing DIO-AAV-ChR2-EYFP exhibited a significant attenuation of cocaine/blue-light preference relative to controls (Lobo, 2010), implicating a role for the activation of D1-expressing MSNs in enhancing the rewarding effects of cocaine, with activation of D2-expressing MSNs antagonizing the cocaine reward effect. Inhibition of D1-expressing MSNs with the tetanus toxin (Hikida et al., 2010) resulted in a diminished cocaine CPP, while no alterations to cocaine CPP after abolishing synaptic transmission in D2-expressing MSNs were observed (Hikida et al., 2010). Therefore, these data using optogenetics and cell-type specific inactivation of neurons implicate opposing roles of D1-and D2-expressing MSNs in CPP, with D1 receptor-expressing MSNs implicated in promoting both reward responses to psychostimulants, and D2 receptor-expressing MSNs dampening these behaviors (Lobo and Nestler, 2011).

### COCAINE SELF-ADMINISTRATION AND COCAINE-SEEKING BEHAVIORS

Cocaine self-administration is an operant model in which laboratory animals lever press (or nose poke) for drug injections. The “self-administration” behavioral paradigm serves as an animal behavioral model of the human pathology of addiction (Thomas et al., 2008). It has been reported that selective lesion of DA terminals with 6-hydroxy DA (6-OHDA), or with the neurotoxin kainic acid in the NAc significantly attenuates cocaine self-administration, supporting the hypothesis that the reinforcing effects of cocaine are dependent upon mesolimbic DA (Pettit et al., 1984; Zito et al., 1985; Caine and Koob, 1994). Consistent with these findings, *in vivo* microdialysis studies demonstrate that accumbal extrasynaptic DA levels are enhanced during cocaineself-administration in both the rat (Hurd et al., 1989; Pettit and Justice, 1989) and monkey (Czoty et al., 2000). Collectively, these findings suggest that enhanced DA transmission in the NAc plays a crucial role in cocaine self-administration behavior.

DA receptor antagonists and agonists modulate cocaine self-administration, showing a dose-dependent biphasic effect. For example, selective antagonists for both D1 (Woolverton, 1986; Britton et al., 1991; Hubner and Moreton, 1991; Vanover et al., 1991; Caine and Koob, 1994) and D2 (Woolverton, 1986; Britton et al., 1991; Hubner and Moreton, 1991; Caine and Koob, 1994) receptors increase cocaine self-administration in response to lower doses of antagonist, but decrease self-administration in response to higher doses. This modulation appears to be specific when injected into the NAc but not the caudate nucleus, indicating a distinct role of NAc DA receptors in cocaine self-administration behaviors.

Later, using D1 and D2 receptor null mice, the involvement of these receptors in the cocaine self-administration was examined. Interestingly, despite the observation of normal cocaine CPP in D1 receptor KO mice, cocaine self-administration was eliminatedin these mice (Caine et al., 2007). In D2 receptor KO mice however, self-administration of low to moderate doses of cocaine was unaffected, while self-administration of moderate to high doses of cocaine was actually increased (Caine et al., 2002). Recently, Alvarez and co-workers reported that synaptic strengthening onto D2-expressing MSNs in the NAc occurs in mice with a history of intravenous cocaine self-administration (Bock et al., 2013). Inhibition of D2-MSNs using a chemicogenetic approach enhanced the motivation to obtain cocaine, while optogenetic activation of D2-MSNs suppressed cocaine self-administration, suggesting that recruitment of D2-MSNs in the NAc functions to restrain cocaine self-administration (Bock et al., 2013).

Studies investigating the reinstatement of cocaine-seeking behavior revealed that the administration of D2 receptor agonists reinstates cocaine-seeking behavior (Self et al., 1996; De Vries et al., 1999, 2002; Spealman et al., 1999; Khroyan et al., 2000; Fuchs et al., 2002). Consistent with these findings, D2 receptor antagonists attenuate cocaine priming-induced drug-seeking behavior (Spealman et al., 1999; Khroyan et al., 2000), while pre-treatment with a D2-like agonist prior to a priming injection of cocaine potentiated the behavior (Self et al., 1996; Fuchs et al., 2002). However, it appears that D1-like receptor agonists do not reinstate cocaine-seeking behavior (Self et al., 1996; De Vries et al., 1999; Spealman et al., 1999; Khroyan et al., 2000). In fact, systemically administered D1-like agonists and antagonists both attenuate the drug-seeking behavior induced by a priming cocaine injection (Self et al., 1996; Norman et al., 1999; Spealman et al., 1999; Khroyan et al., 2000, 2003), showing a differential involvement of D1 and D2 receptors in priming-induced reinstatement of cocaine seeking.

Results from our laboratory indicate that in the absence of D2 receptors, cocaine-induced reinstatement was not affected (Sim et al., 2013). It is suggested that the reinstatement of drug-seeking behavior can also be precipitated by re-exposure to cocaine-associated stimuli or stressors (Shaham et al., 2003). When this possibility was tested, results from our laboratory found that while stress potentiates the cocaine-induced reinstatement in WT mice, stress suppressed the cocaine-induced reinstatement in the D2 receptor mutant animals, suggesting an unexplored role of D2 receptors in the regulation of synaptic modification triggered by stress and drug addiction (Sim et al., 2013).

## DOPAMINE SIGNALING IN FOOD REWARD

Food and food-related cues can activate different brain circuits involved in reward, including the NAc, hippocampus, amygdala and/or pre-frontal cortex and midbrain (Palmiter, 2007; Kenny, 2011). It is believed that the mesolimbic DA system promotes the learning of associations between natural reward and the environments in which they are found; thus, food and water, or cues that predict them, promote rapid firing of DA neurons, and facilitate behaviors directed toward acquisition of the reward (Palmiter, 2007). Indeed DA-deficient mice show a loss of motivation to feed (Zhou and Palmiter, 1995), while D1 receptor null mice exhibit retarded growth and low survival after weaning; this phenotype can be rescued by providing KO mice with easy access to a palatable food, suggesting that the absence of D1 receptor is more related to a motor deficit (Drago et al., 1994; Xu et al., 1994). In contrast, D2 receptor KO mice show reduced food intake and body weight along with an increased basal energy expenditure level compared to their wild type littermates (Kim et al., 2010). Therefore, it is difficult to delineate the exact role of the DA system and of the receptor subtypes in food reward. Nevertheless, most human studies indicate the importance of the D2 receptor in the regulation of food reward in association with obesity.

### D2 RECEPTOR EXPRESSION IN FOOD REWARD

Increasing evidence suggests that variations in DA receptors and DA release play a role in overeating and obesity, especially in association with striatal D2 receptor function and expression (Stice et al., 2011; Salamone and Correa, 2013). In animal studies, it has been shown that feeding increases the extracellular DA concentration in the NAc (Bassareo and Di Chiara, 1997), in a similar manner to drugs of abuse. However, in contrast to its effect on behaviors related to drug addiction, NAc DA depletion alone does not alter feeding behavior (Salamone et al., 1993). It appears that the pharmacological blockade of D1 and D2 receptors in the NAc affects motor behavior, amount and duration of feeding, but it does not reduce the amount of food consumed (Baldo et al., 2002). Interestingly, recent data showed that binge eating was ameliorated by the acute administration of unilateral NAc shell deep brain stimulation, and this effect was mediated in part by activation of the D2 receptor, while deep brain stimulation of the dorsal striatum had no influence on this behavior (Halpern et al., 2013) in mice. However, it has been reported that when exposed to the same high-fat diet, mice with a lower density of D2 receptors in the putamen exhibit more weight gain than mice with a higher density of D2 receptorsin the same region (Huang et al., 2006). This study compared DAT and D2 receptor densities in chronic, high-fat diet-induced obese, obese-resistant and low-fat-fed control mice, and found that D2 receptor density was significantly lower in the rostral part of caudate putamen in chronic high-fat diet-induced obese mice compared to obese-resistant and low-fat-fed control mice (Huang et al., 2006). This low level of D2 receptor may be associated with altered DA release, and it has also been reported that consumption of a high-fat, high-sugar diet leads to the downregulation of D2 receptors (Small et al., 2003) and reduced DA turnover (Davis et al., 2008).

In human studies, obese people and drug addicts both tend to show reduced expression of D2 receptors in striatal areas, and imaging studies have demonstrated that similar brain areas are activated by food- and drug-related cues (Wang et al., 2009). Positron emission tomography (PET) studies suggest that the availability of D2 receptors was decreased in obese individuals in proportion to their body mass index (Wang et al., 2001), thus suggesting that DA deficiency in obese individuals may perpetuate pathological eating as a means of compensating for the decreased activation of DA-mediated reward circuits. Volkow and co-workers also reported that obese versus lean adults show less striatal D2 receptor binding, and that this was positively correlated with metabolism in the dorsolateral prefrontal, medial orbitofrontal, anterior cingulate gyrus and somatosensory cortices (Volkow et al., 2008). This observation led to a discussion over whether decreases in striatal D2 receptors could contribute to overeating via the modulation of striatal prefrontal pathways that participate in inhibitory control and salience attribution, and whether the association between striatal D2 receptors and metabolism in the somatosensory cortices (regions that process palatability) could underlie one of the mechanisms through which DA regulates the reinforcing properties of food (Volkow et al., 2008).

Stice and co-workers used functional magnetic resonance imaging (fMRI) to show that individuals may overeat to compensate for a hypofunctioning dorsal striatum, particularly those with genetic polymorphisms of an A1 allele of the TaqIA in D2 receptor (*DRD2/ANKK1)* gene, which is associated with lower striatal D2 receptor density and attenuated striatal DA signaling (Stice et al., 2008a,b). These observations indicate that individuals who show blunted striatal activation during food intake are at risk for obesity, particularly those also at genetic risk for compromised DA signaling in brain regions implicated in food reward (Stice et al., 2008a, 2011). However, recent data showed that obese adults with or without binge eating disorder had a distinct genetic polymorphism of the TaqIA D2 receptor (*DRD2/ANKK1*) gene (Davis et al., 2012); therefore, it is plausible that similar brain DA systems are disrupted in both food motivation and drug addiction, even though it is not yet clear what these DA receptor data represent from the functional perspective of DA neurotransmission in brain.

As in obese people, low D2 receptor availability is associated with chronic cocaine abuse in humans (Volkow et al., 1993; Martinez et al., 2004). In contrast, overexpression of D2 receptors reduces the self-administration of alcohol in rats (Thanos et al., 2001). In humans, a higher-than-normal D2 receptor availability in non-alcoholic members of alcoholic families was reported (Volkow et al., 2006; Gorwood et al., 2012), supporting the hypothesis that low levels of D2 receptors may be associated with an increased risk of addictive disorders. Therefore, it is possible that in the brains of both obese individuals and chronic drug abusers, there are low basal DA concentrations, and periodic exaggerated DA release associated with either food or drug intake, along with low expression, or dysfunctional D2 receptors.

Dopamine receptor expression levels in other areas of the brain may also be important. For example, Fetissov et al. (2002) observed that obese Zucker rats, which display a feeding pattern consisting of large meal size and small meal number, have a comparatively low level of D2 receptor expression in the ventromedial hypothalamus (VMH). Interestingly, in their study, when a selective D2 receptor antagonist, sulpiride was injected into the VMH of obese and lean rats, a hyperphagic response was elicited only in the obese rats, suggesting that by aggravating the already low level of D2 receptors, it was possible to increase food intake. This low D2 receptor expression may cause an exaggerated DA release in obese rats during food ingestion and a reduced satiety feedback effect of DA, which would facilitate DA release into the brain areas “craving” for DA (Fetissov et al., 2002).

Recently, in an elegant study conducted by Johnson and Kenny (2010), it was observed animals provided with a “cafeteria diet” consisting of a selection of highly palatable energy-dense food gained weight, demonstrating compulsive eating behavior. In addition to their excessive adiposity and compulsive-like eating, cafeteria diet rats also had decreased D2 receptor expression in the striatum. Surprisingly, lentivirus-mediated knockdown of striatal D2 receptors rapidly accelerated the development of addiction-like reward deficits, and the onset of compulsive-like food-seeking behaviorin rats with extended access to palatable high-fat food (Johnson and Kenny, 2010), again indicating that common hedonic mechanisms may therefore underlie obesity and drug addiction. However, our own laboratory found somewhat unexpected results showing that D2 KO mice have a lean phenotype with enhanced hypothalamic leptin signaling compared to WT mice (Kim et al., 2010). Therefore, we cannot rule out that the D2 receptor plays a role in the homeostatic regulation of metabolism in association with a regulator of energy homeostasis such as leptin, in addition to its role in food motivation behavior. An animal model with a genetically manipulated conditional restriction of the D2 receptor in leptin receptor-expressing cells for example, or other reward-related neuronal cells, together with neural integrative tools, could potentially elucidate the role of the DA system via D2 receptors in food reward and the homeostatic regulation of food intake.

### DOPAMINERGIC REWARD SIGNALING LINKED TO HOMEOSTATIC FEEDING CIRCUIT

Increasing evidence indicates that homeostatic regulators of food intake, such as leptin, insulin, and ghrelin, control and interact with the reward circuit of food intake, and thus regulate behavioral aspects of food intake and conditioning to food stimuli behaviors (Abizaid et al., 2006; Fulton et al., 2006; Hommel et al., 2006; Baicy et al., 2007; Farooqi et al., 2007; Palmiter, 2007; Konner et al., 2011; Volkow et al., 2011). Recent findings reveal that hormones implicated in regulating energy homeostasis also impinge directly on DA neurons; for example, leptin and insulin directly inhibit DA neurons, while ghrelin activates them (Palmiter, 2007; Kenny, 2011).

Hommel and co-workers demonstrated that VTA DA neurons express leptin receptor mRNA, and respond to leptin with the activation of an intracellular JAK-STAT (Janus kinase-signal transducer and activator of transcription) pathway, which is the major pathway involved in leptin receptor downstream signaling, as well as a reduction in the firing rate of DA neurons (Hommel et al., 2006). This study showed that direct administration of leptin to the VTA caused decreased food intake, while long-term RNAi-mediated knockdown of leptin receptors in the VTA led to increased food intake, locomotor activity, and sensitivity to highly palatable food. These data support a critical role for VTA leptin receptorsin regulating feeding behavior, and provide functional evidence for the direct action of a peripheral metabolic signal on VTA DA neurons. These results are consistent with the idea that leptin signaling in the VTA normally suppresses DA signaling, and consequently decreases both food intake and locomotor activity. This suggests a physiological role for leptin signaling in the VTA, although the authors did not demonstrate that the effect of the virus injection on feeding was correlated directly with increased DA signaling (Hommel et al., 2006).

Fulton and co-workers also investigated the functional significance of leptin action in VTA DA neurons, to expand understanding of the multiple actions of leptin in the DA reward circuit (Fulton et al., 2006). Using double-label immunohistochemistry, they observed increased STAT3 phosphorylation in the VTA following peripheral leptin administration. These pSTAT3-positive neurons colocalized with DA neurons, and to a lesser extent with markers for GABA neurons. Retrograde neuronal tracing from the NAc revealed colocalization of the tracer with pSTAT3, indicating that a subset of VTA DA neurons expressing leptin receptors project to the NAc. When they assessed leptin function in the VTA, they found that *ob/ob* mice had a diminished locomotor response to amphetamine, and lacked locomotor sensitization to repeated amphetamine injections, with both defects being reversed by leptin infusion, thus indicating that the mesoaccumbens DA pathway, critical to integrating motivated behavior, also responds to this adipose-derived signal (Fulton et al., 2006). These lines of evidence importantly suggested the action of leptin in the DA reward system. However, given that physiological level of leptin receptor expression appear to be very low in the midbrain, normal circulating leptin levels seem to have little effect on leptin receptor signaling within the VTA. Thus, whether *in vivo* leptin can exert an significant effect to inhibit DA neuron activity through their receptors in VTA remains questionable (Palmiter, 2007).

There are also human studies showing that leptin can indeed control rewarding responses. Farooqi and co-workers reported that patients with congenital leptin deficiency displayed activation of DA mesolimbic targets (Farooqi et al., 2007). In the leptin-deficient state, images of well-liked foods engendered a greater wanting response, even when the subject has just been fed, while after leptin treatment, well-liked food images engendered this response only in the fasted state, an effect consistent with the response in control subjects. Leptin reduces activation in the NAc-caudate, and mesolimbic activation (Farooqi et al., 2007). Thus, this study suggests that leptin diminished the rewarding responses to food, acting on the DA system (Farooqi et al., 2007; Volkow et al., 2011). Another fMRI study by Baicy et al., also performed with patients with congenital leptin deficiency, showed that during viewing of food-related stimuli, leptin replacement reduced neural activation in brain regions linked to hunger (the insula, parietal and temporal cortex), while enhancing activation in regions linked to inhibition and satiety (the prefrontal cortex; Baicy et al., 2007). Therefore, it appears that leptin acts on neural circuits involved in hunger and satiety with inhibitory control.

Another peptide hormone, ghrelin, which is produced in the stomach and pancreas, is known to increase appetite and food intake (Abizaid et al., 2006). The ghrelin receptor growth hormone secretagogue 1 receptor (GHSR) is present in hypothalamic centers as well as in the VTA. Abizaid and co-workers showed that in mice and rats, ghrelin bound to neurons of the VTA, where it triggered increased DA neuronal activity, synapse formation, and DA turnover in the NAc, in a GHSR-dependent manner. In addition, they demonstrated that direct VTA administration of ghrelin also triggered feeding behavior, while intra-VTA delivery of a selective GHSR antagonist blocked the orexigenic effect of circulating ghrelin, and blunted rebound feeding following fasting, suggesting that the DA reward circuitry is targeted by ghrelin to influence motivation for food (Abizaid et al., 2006).

Insulin, which is one of the key hormones involved in the regulation of glucose metabolism, and inhibits feeding, has been shown to also regulate the DA system in the brain. Insulin receptors are expressed in brain regions that are rich in DA neurons, such as the striatum and midbrain (Zahniser et al., 1984; Figlewicz et al., 2003), suggesting a functional interaction between the insulin and DA systems. Indeed, it has been shown that insulin acts on DA neurons, and infusion of insulin into the VTA decreases food intake in rats (Figlewicz et al., 2008; Bruijnzeel et al., 2011). Recent studies on the selective deletion of insulin receptors in midbrain DA neurons in mice demonstrated that this manipulation results in increased body weight, increased fat mass, and hyperphagia (Konner et al., 2011). While insulin acutely stimulated firing frequency in 50% of dopaminergic VTA/SN neurons, this response was abolished in those mice with the insulin receptor selectively deleted in DA neurons. Interestingly, in these mice, D2 receptor expression in the VTA was decreased compared to control mice. Moreover, these mice exhibited an altered response to cocaine under food-restricted conditions (Konner et al., 2011). Another recent report indicates that insulin can induce long-term depression (LTD) of mouse excitatory synapses onto VTA DA neurons (Labouèbe et al., 2013). Furthermore, after a sweetened high-fat meal, which elevates endogenous insulin levels, insulin-induced LTD is occluded. Finally, insulin in the VTA reduces food anticipatory behavior in mice, and CPP for food in rats. This study raises an interesting issue about how insulin can modulate reward circuitry, and suggests a new type of insulin-induced synaptic plasticity on VTA DA neurons (Labouèbe et al., 2013).

## CONCLUSIONS AND FUTURE DIRECTIONS

This review has focused on the role of the DA system, mainly concentrating on the roles of D1 and D2 receptors in reward-related behaviors, including addiction and food motivation. However, it is well known that the DA system in this reward-circuit is finely modulation by glutamatergic, GABAergic, and other neurotramistter systems, which form specific circuits to encode the neuronal correlates of behaviors. Recent breakthroughs in optogenetic tools to alter neuronal firing and function with light, as well as DREADDs, together with genetic manipulation of specific neuronal cells or circuits are now allowing us to refine our insight into reward circuits in addiction, and the hedonic value of food intake. It is of no doubt that these lines of investigation have provided a foundation for future direction of our study in neurocircuitry of the DA system in these behaviors. Future studies could include enlarged manipulations of important signaling molecules, for example, signaling molecules implicated in the D1 and D2 receptor signaling cascades, to explore the impact of these molecules on the induction and expression of specific reward behaviors. Given that these two receptors employ distinct signaling pathways, in terms of their respective G protein coupling, as well as in the activation of common singling molecules such as ERK, the differential distribution of receptors, as well as of their downstream signaling molecules may result in a different type of physiological response. Additionally, with this conceptual and technical evolution of the DA system in behaviors, this research will have important implications in the clinical investigation of related neurological disorders and psychiatric diseases. Therefore, our continuing efforts to identify and characterize the organization and modification of DA synaptic functions in both animals and humans will contribute to the elucidation of neural circuits underlying the pathophysiology of drug addiction and eating disorders.

## Conflict of Interest Statement

The author declares that the research was conducted in the absence of any commercial or financial relationships that could be construed as a potential conflict of interest.
